# Family Caregiver Health in a Pandemic

**DOI:** 10.1093/geroni/igab046.3602

**Published:** 2021-12-17

**Authors:** Kimberly Cassie

**Affiliations:** University of Oklahoma, Tulsa, Oklahoma, United States

## Abstract

Each year family caregivers provide care and services worth billions of dollars to support the needs of older Americans. Their support is invaluable to keep individuals in the community for as long as possible and to allow individuals to attain and maintain their highest practicable level of well-being. But what impact does caregiving have on one’s health? Does caregiver health decline with the assumption of caregiving duties? Did caregiver health change during the pandemic? If so, how and what factors are associated with declines in caregiver health? To answer these questions, an exploratory survey was conducted among a convenience sample of 195 family caregiver. Almost a third of those sampled reported excellent or very good health, while 44% reported good health, and almost a quarter reported poor or fair health. Forty-eight percent reported their health had declined since they assumed caregiving duties and 29% reported their health had declined during the pandemic. Employed caregivers and those experiencing less depression/anxiety reported better health. Those experiencing a decline in health with caregiving were more likely to be female, not employed, experienced more stress and more depression/anxiety. Those experiencing a decline in health during the pandemic reported less spirituality, greater attachment related avoidance, and greater depression/anxiety. Findings from this research can be used to inform future research on the effect of the pandemic on family caregiving and to plan interventions to protect caregiver health as they provide vital services to maintain individuals in the community for as long as possible.

